# Hinokitiol Inhibits Breast Cancer Cells In Vitro Stemness-Progression and Self-Renewal with Apoptosis and Autophagy Modulation via the CD44/Nanog/SOX2/Oct4 Pathway

**DOI:** 10.3390/ijms25073904

**Published:** 2024-03-31

**Authors:** Yi-Fen Chiang, Ko-Chieh Huang, Hsin-Yuan Chen, Nadia M. Hamdy, Tsui-Chin Huang, Hsin-Yi Chang, Tzong-Ming Shieh, Yun-Ju Huang, Shih-Min Hsia

**Affiliations:** 1 School of Nutrition and Health Sciences, College of Nutrition, Taipei Medical University, Taipei 110301, Taiwan; da07108002@tmu.edu.tw (Y.-F.C.); da07111001@tmu.edu.tw (K.-C.H.); d507104002@tmu.edu.tw (H.-Y.C.); 2 Biochemistry Department, Faculty of Pharmacy, Ain Shams University, Abassia, Cairo 11566, Egypt; nadia_hamdy@pharma.asu.edu.eg; 3 Graduate Institute of Cancer Biology and Drug Discovery, College of Medical Science and Technology, Taipei Medical University, Taipei 110301, Taiwan; tsuichin@tmu.edu.tw; 4 Graduate Institute of Medical Science, National Defense Medical Center, Taipei 11490, Taiwan; hsinyi.chang@mail.ndmctsgh.edu.tw; 5 School of Dentistry, College of Dentistry, China Medical University, Taichung 40402, Taiwan; 6 Department of Biotechnology and Food Technology, Southern Taiwan University of Science and Technology, Tainan City 710301, Taiwan; yjhuang@stust.edu.tw; 7 School of Food and Safety, Taipei Medical University, Taipei 110301, Taiwan; 8 Nutrition Research Center, Taipei Medical University Hospital, Taipei 110301, Taiwan; 9 Graduate Institute of Metabolism and Obesity Sciences, College of Nutrition, Taipei Medical University, Taipei 110301, Taiwan; 10 TMU Research Center for Digestive Medicine, Taipei Medical University, Taipei 110301, Taiwan

**Keywords:** breast cancer stem cell, hinokitiol, apoptosis, autophagy, CD44

## Abstract

Breast cancer (BC) represents one of the most prevalent malignant threats to women globally. Tumor relapse or metastasis is facilitated by BC stemness progression, contributing to tumorigenicity. Therefore, comprehending the characteristics of stemness progression and the underlying molecular mechanisms is pivotal for BC advancement. Hinokitiol (β-thujaplicin), a tropolone-related compound abundant in the heartwood of cupressaceous plants, exhibits antimicrobial activity. In our study, we employed three BC cell lines (MDA-MB-231, MCF-7, and T47D) to assess the expression of stemness-, apoptosis-, and autophagy-related proteins. Hinokitiol significantly reduced the viability of cancer cells in a dose-dependent manner. Furthermore, we observed that hinokitiol enhances apoptosis by increasing the levels of cleaved poly-ADP-ribose polymerase (PARP) and phospho-p53. It also induces dysfunction in autophagy through the upregulation of LC3B and p62 protein expression. Additionally, hinokitiol significantly suppressed the number and diameter of cancer cell line spheres by reducing the expression of cluster of differentiation44 (CD44) and key transcription factors. These findings underscore hinokitiol’s potential as a therapeutic agent for breast cancer, particularly as a stemness-progression inhibitor. Further research and clinical studies are warranted to explore the full therapeutic potential of hinokitiol in the treatment of breast cancer.

## 1. Introduction

Breast cancer (BC) is a highly lethal disease that poses a significant global health challenge [1], with approximately 2.3 million new cases diagnosed each year worldwide [2]. It can originate from various parts of the breast tissue, including the milk ducts, lobules, and even the connective tissue, contributing to its heterogeneity [3]. BC is classified into different subtypes based on its molecular features, including luminal A, luminal B, human epidermal growth factor receptor-2 (HER2/erb2) positive, and the most aggressive type, triple-negative breast cancer (TNBC) [4]. Luminal A BC is the most common subtype and is characterized by the presence of estrogen receptor (ER) and progesterone receptor (PR) expression. Patients with luminal A BC typically have a more favorable prognosis and respond well to endocrine therapy, making it a subtype with better treatment outcomes [5]. The most malignant BC type is TNBC, which accounts for 10–15% of all BC cases, with high resistance to first line chemotherapy treatment [6]. One contributing factor to chemotherapy resistance and cancer progression is the phenomenon of “stemness”, a biological process involved in cancer cell self-renewal [7,8]. Cancer metastatic progression occurs via stemness-induced cancer cell differentiation and tumor proliferation-related migration [9,10].

During cancer stemness progression, stemness-related proteins play pivotal roles as major modulators and surface markers, contributing to cancer cell self-renewal [11]. The cluster of differentiation44 (CD44) as a stemness-related protein is associated with cancer stem cell maintenance in BC, being involved in cells adhesion, cell-to-cell interaction, tumor cells migration-for-metastasis as well as signal transduction pathways [12]. In addition to CD44, Nanog Homeobox (Nanog), SRY (Sex Determining Region Y)-Box 2 (SOX2), and Octamer-Binding Transcription Factor 4 (Oct4) play crucial roles in maintaining cancer stemness progression and the poor prognoses in cancer patients [13].

P53, a common tumor suppression modulator, can inhibit cancer cell proliferation and cancer progression. The absence of the TP53 gene (p53) expression also enhances the resistance of cancer cells to apoptosis progression, leading to the inactivation of poly(ADP-ribose) polymerase-1 (PARP-1) [14]. The induction of apoptosis, as part of the p53-dependent apoptosis response to DNA damage, plays a crucial role in the management and treatment of tumor suppression [15].

Hinokitiol (beta-thujaplicin, 4-isopropyltropolone) is one of the natural compounds separated from heartwood of Chamaecyparis obtusa and Thuja plicata. Hinokitiol is a monoterpenoid beta-thujaplicin with a hydroxy group substituted at position 2 and an isopropyl group substituted at position 4 (2-hydroxy-4-isopropyl- 2,4,6-cycloheptatriene-1-one) or 4-isopropyltropolone or beta-thujaplicin. Hinokitiol has anti-inflammatory, antioxidant, and anti-microbial properties [16,17]. Moreover, hinokitiol neuroprotective effect against amyloid-beta-induced toxicity was related to its anti-inflammatory and antioxidant ability to reduce reactive oxygen species (ROS) [18]. In light of these previous effects, several studies had examined hinokitiol’s anti-cancer ability to induce apoptosis with cell cycle arrest or achieving angiogenesis reduction via specific signal transduction pathways inhibition [19,20,21,22]. Hinokitiol has been shown to suppress androgen-stimulated DNA synthesis, leading to the suppression of prostate cancer [23] and the reduction of estrogen receptor α expression, resulting in the suppression of breast cancer [24]. Additionally, the Fe complex has been found to accelerate ferroptosis and reduce breast cancer progression [25]. The inhibition of the Akt/mTOR pathway could modulate the autophagy progression [26]. Moreover, in the melanoma model, hinokitiol exhibited the inhibition of mitogen-activated protein kinase (MAPK) signaling pathway [27]. Therefore, in the current study, we aimed to explore the hinokitiol’s anti-BC molecular mechanism(s) via stemness progression inhibition, with apoptosis and autophagy induction mechanisms, a step toward approving the pre-clinical in vitro hinokitiol efficiency for BC treatment.

Here, we used three different cell lines. MDA-MB-231 represents the aggressive triple-negative subtype of breast cancer, which is known for its poor prognosis and limited treatment options. MCF-7 and T47D cells serve as representative models of breast cancer with a less aggressive phenotype, allowing us to compare the effects of the hinokitiol across different breast cancer subtypes.

In this study, our aim was to examine hinokitiol’s potential to modulate the viability of three BC cell lines at different doses, assess its induction of apoptosis, and evaluate its impact on autophagy-related proteins. Additionally, we sought to further explore hinokitiol’s modulation of sphere formation and elucidate the related molecular mechanisms.

## 2. Results

### 2.1. Hinokitiol on Breast Cancer Cell Viability

**Figure 1:**
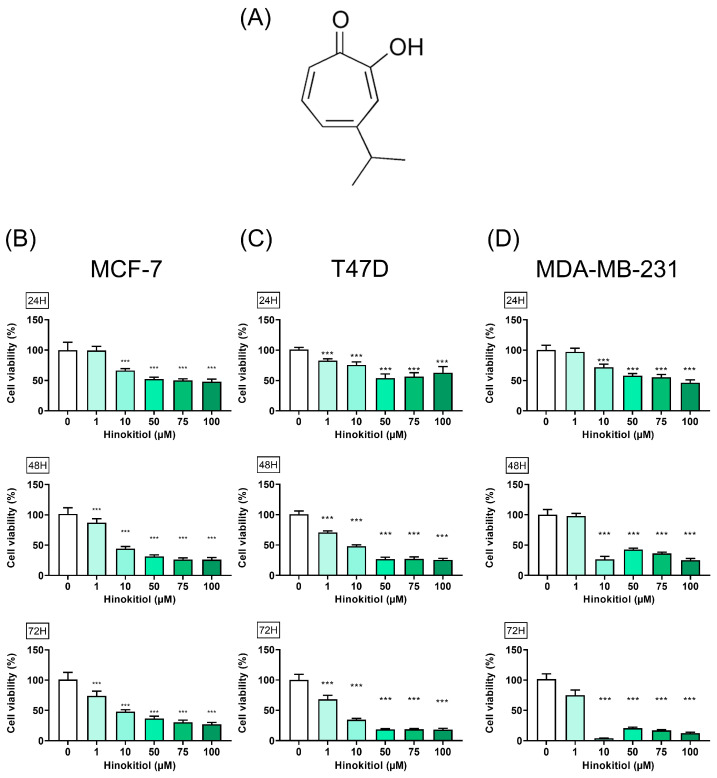
Effect of hinokitiol on breast cancer cell viability. (**A**) Hinokitiol structure, (**B**) MCF-7, (**C**) T47D, and (**D**) MDA-MB-231 cells were cultured in 96 well plate (3000 cells/well) and treated with hinokitiol for 24, 48, and 72 h. MTT assay was used to determine cell viability. Data are presented as mean ± SD (n = 5–6). ***, *p* < 0.001 as compared with control group.

To assess the potential anti-cancer effect of hinokitiol on breast cancer cells, we conducted experiments on MCF-7 (Figure 1A), T47D (Figure 1B), and MDA-MB-231 (Figure 1C) cell lines. Cells were treated with varying concentrations of hinokitiol (0, 1, 10, 50, 75, and 100 μM) for 24, 48, and 72 h. Cell viability was determined using the MTT assay, which measures the ability of viable cells to convert MTT to a purple formazan product. The results showed that hinokitiol treatment led to a dose- and time-dependent inhibition of breast cancer cell viability, with low cytotoxicity up to 200 μM in normal cell line [28]. These findings suggest that hinokitiol has the potential to modulate the viability of breast cancer cells.

### 2.2. Hinokitiol Enhanced Breast Cancer Cell Apoptosis via Increasing Cleaved-PARP and p-p53 Protein Expression

**Figure 2:**
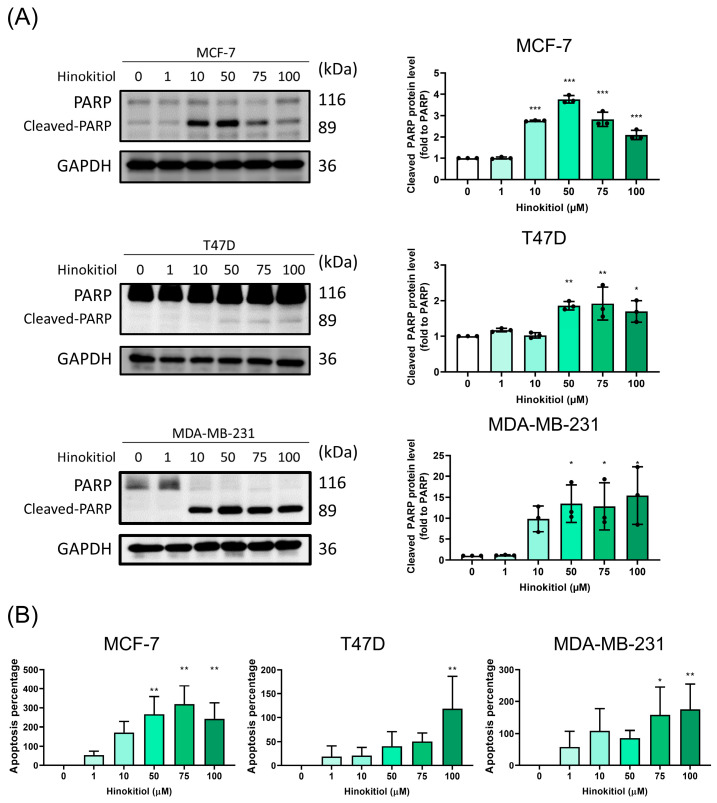
Effect of hinokitiol on apoptosis progression in breast cancer MCF-7, T47D, and MDA-MB-231 cells were cultured in a 6 cm dish and treated with hinokitiol for 48 h. Western blot was used to determine (**A**) PARP protein expression and (**B**) automated fluorescence cell counter ADAM-MC was used to measure the apoptosis percentage. Data are presented as mean ± SD (n = 3–4). * *p* < 0.05; ** *p* < 0.01; *** *p* < 0.001 as compared with control group.

**Figure 3:**
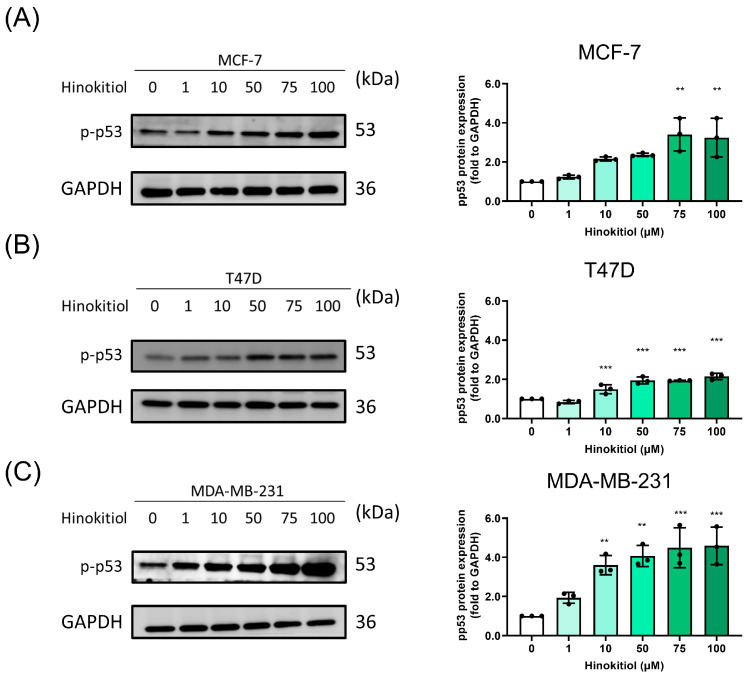
Effect of hinokitiol on tumor suppressor p-p53 protein expression. (**A**) MCF-7, (**B**) T47D, and (**C**) MDA-MB-231 cells were cultured in a 6 cm dish and treated with hinokitiol for 48 h. Western blot was used to determine p-p53 protein expression. Data are presented as mean ± SD (n = 3). ** *p* < 0.01; *** *p* < 0.001 as compared with control group.

Apoptosis serves as a key mechanism for modulating cancer suppression, involving a programmed cell death process to eliminate abnormal cells. In investigating the potential mechanisms underlying the anti-proliferative effects of hinokitiol on breast cancer cells, namely MCF-7, T47D, and MDA-MB-231 cells, we subjected them to hinokitiol treatment for 48 h. This treatment resulted in a significant increase in cleaved-PARP (as shown in Figure 2A) and induced apoptosis percentages (as depicted in Figure 2B) across all three breast cancer cell lines. Furthermore, flow cytometry analysis indicated that hinokitiol could enhance apoptosis percentages in the TNBC cell line, as demonstrated in Supplementary Figure S1. The activation of p53, a tumor suppressor, is known to induce apoptosis in breast cancer cells. Our assessment of p-p53 protein expression revealed that hinokitiol treatment significantly upregulated p-p53 when compared to the control group (Figure 3). This upregulation of cleaved-PARP and p-p53 protein expression underscores hinokitiol’s ability to modulate apoptosis, thereby enhancing apoptosis in breast cancer cells.

### 2.3. Modulation of Hinokitiol in Autophagy Influx

**Figure 4:**
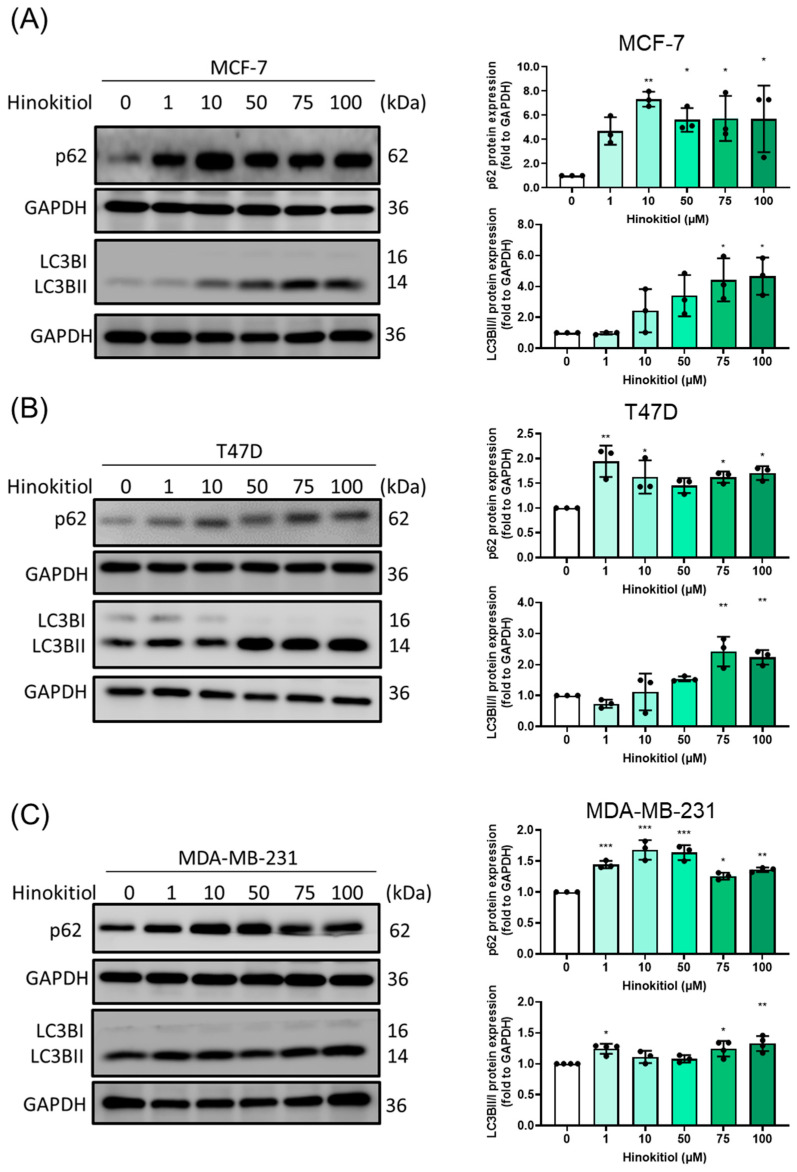
Effect of hinokitiol on autophagy-related protein expression. (**A**) MCF-7, (**B**) T47D, and (**C**) MDA-MB-231 cells were cultured in a 6 cm dish and treated with hinokitiol for 48 h. Western blot was used to determine p62 and LC3BII/I protein expression. Data are presented as mean ± SD (n = 3–4). * *p* < 0.05; ** *p* < 0.01; *** *p* < 0.001 as compared with control group.

Apoptosis and autophagy influx were critical for anti-cancer effect. Autophagy is the procedure that could eliminate the unnecessary cellular components. The modulation of autophagy was related to cancer progression and chemotherapy. To investigate the potential mechanism underlying the inhibitory effect of hinokitiol on breast cancer stemness, the expression levels of autophagy-related proteins were evaluated in MCF-7, T47D, and MDA-MB-231 cells treated with hinokitiol. With the LC3BII induction and p62 (Figure 4) increase contributed to the autophagy dysfunction indicated the clearance blockage and show the similarity with cisplatin treatment [29]. Autophagy influx was observed to be obstructed, which was associated with an increase in p62 levels and a failure to complete lysosome fusion abilities [30]. In our study, we used the lysosome marker cathepsin B (Supplementary Figure S2) to demonstrate a reduction in lysosomes, indicative of lysosome dysfunction caused by hinokitiol. These findings shed light on the impairment of autophagy-mediated degradation and hint at the potential role of hinokitiol as an adjuvant in breast cancer treatment.

### 2.4. Effect of Hinokitiol on MCF-7, T47D, MDA-MB-231 Breast Cancer Cell Sphere Number and Diameter Analysis

**Figure 5:**
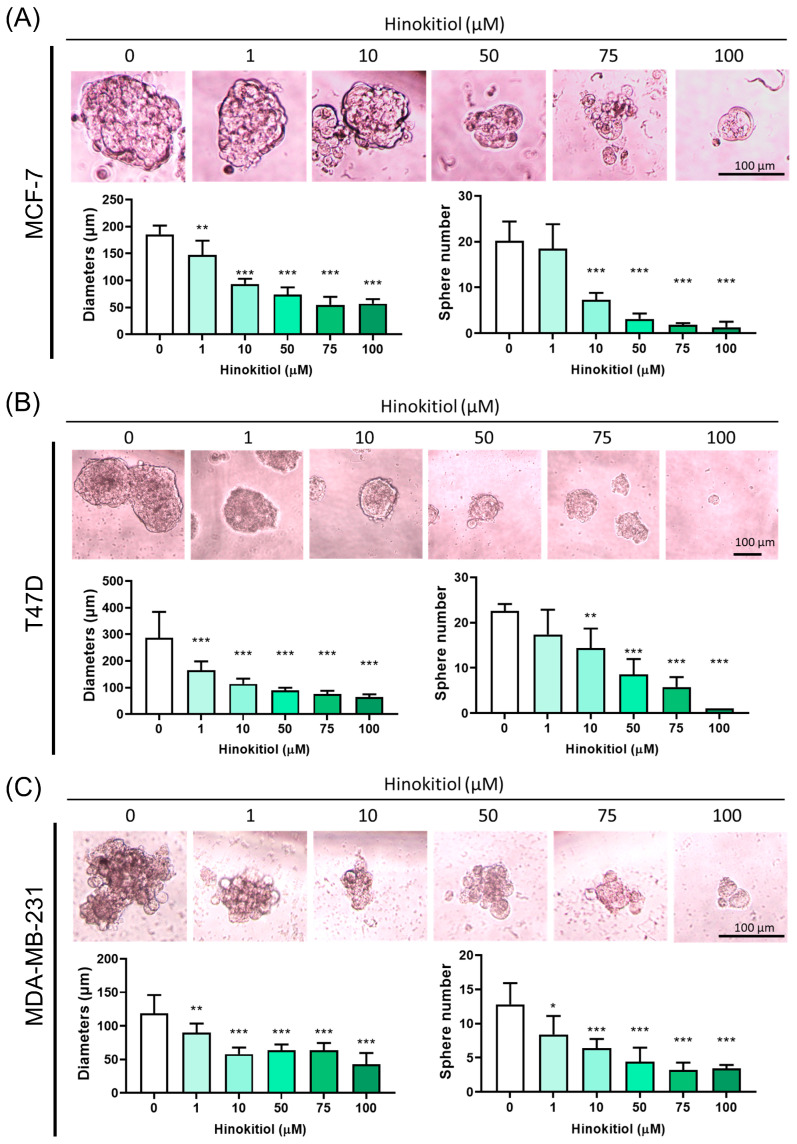
Hinokitiol suppressed sphere formation. (**A**) MCF-7, (**B**) T47D, and (**C**) MDA-MB-231 cells were cultured in a 6 cm dish and treated with hinokitiol for 48 h. After treatment, cells were detached and seeded at a density of 2000 cells per well in ultra-low attachment plates. A sphere formation assay was employed to evaluate both diameters and numbers of spheres formed. Data are presented as mean ± SD (n = 5–6). * *p* < 0.05; ** *p* < 0.01; *** *p* < 0.001 as compared with control group.

To investigate the effect of hinokitiol on stemness progression in breast cancer, MCF-7, T47D, and MDA-MB-231 cells were cultured in a sphere-formation assay that could evaluate the enrichment of stemness progression. The number and diameter of spheres in all three breast cancer cell lines were significantly suppressed by hinokitiol, indicating the inhibition of stemness progression. The reduction of sphere formation suggested the inhibition of self-renewal and differentiation abilities of hinokitiol (Figure 5). These results suggest that hinokitiol has the potential to target stemness progression in breast cancer, which may contribute to its anti-cancer effects.

### 2.5. Effect of Hinokitiol on MCF-7, T47D, MDA-MB-231 Breast Cancer Cell Stemness Related Protein Expression

The evaluation of stemness-related transcription factors has been shown to play critical roles in maintaining stemness properties in cancer cells. Nanog is involved in the regulation of self-renewal and differentiation, while SOX2 and Oct4 are involved in the maintenance of pluripotency in embryonic stem cells. CD44 is a cell surface glycoprotein that plays a critical role in cell adhesion, migration, and invasion. It is also a well-known stem cell marker that has been implicated in the regulation of stemness and the development of cancer stem cells.

**Figure 6:**
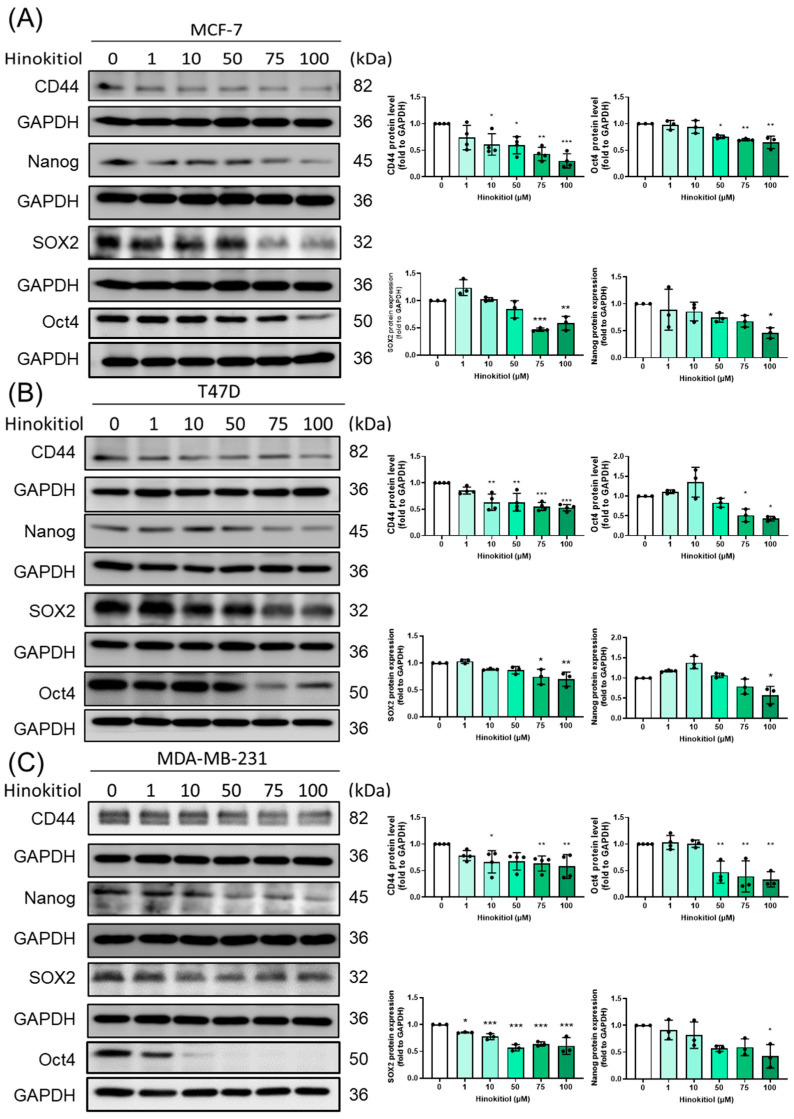
Effect of hinokitiol on stemness-related protein expression. (**A**) MCF-7, (**B**) T47D, and (**C**) MDA-MB-231 cells were cultured in a 6 cm dish and treated with hinokitiol for 48 h. Western blot was used to determine CD44, Nanog, SOX2, and Oct4 protein expression. Data are presented as mean ± SD (n = 3–4). * *p* < 0.05; ** *p* < 0.01; *** *p* < 0.001 as compared with control group.

To investigate the potential mechanism underlying the inhibitory effect of hinokitiol on stemness progression, the expression levels of stemness related protein expression, CD44, Nanog, SOX2, and Oct4 (Figure 6) were evaluated by Western blot.

Hinokitiol treatment significantly reduced CD44 protein expression levels in all three breast cancer cell lines, indicating its potential to inhibit stemness properties and limit the capacity for self-renewal in breast cancer cells. The reduction in Nanog, SOX2, and Oct4 protein expression levels suggests that hinokitiol may inhibit the stemness properties of breast cancer cells by suppressing the key regulators of stemness and the modulation of self-renewal and differentiation in cancer progression.

### 2.6. Effect of Autophagy Modulation in Hinokitiol’s Effect on Proliferation and Stemness Related Protein Expression

**Figure 7:**
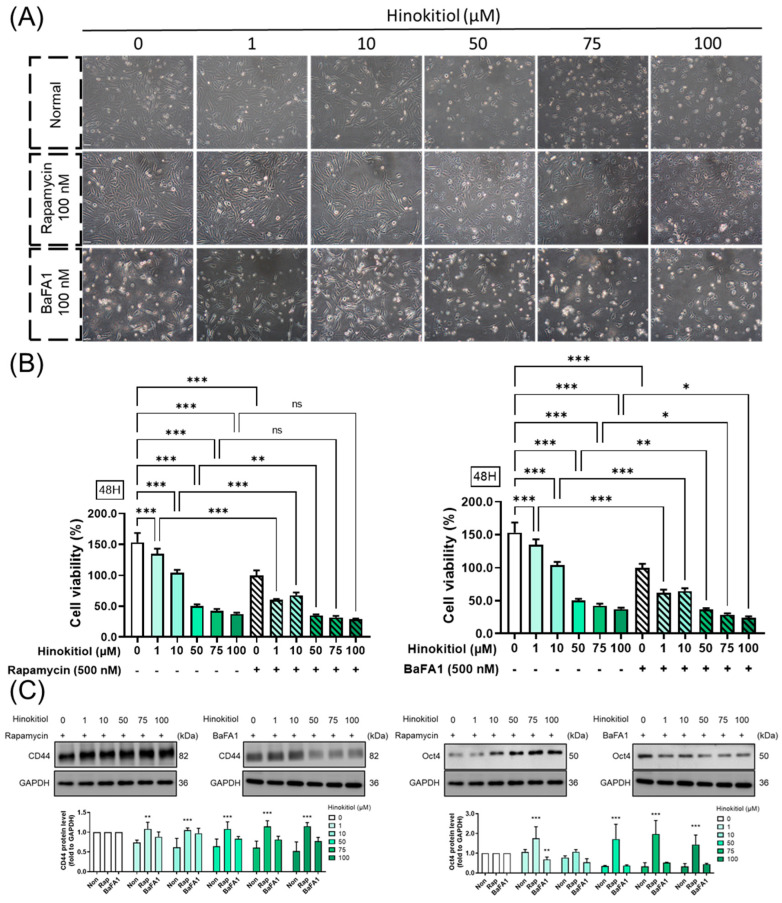
Impact of autophagy modulation on hinokitiol’s effects on proliferation and stemness related protein expression in MDA-MB-231 cells. MDA-MB-231 cells were initially pretreated with rapamycin for 24 h, followed by a 1 h pretreatment with bafilomycin A1 (BaFA1). Subsequently, cells were treated with hinokitiol for 48 h. (**A**) Cell morphology was observed through microscopy, scale bar: 50 μm (**B**) cell viability was assessed using MTT assay, and (**C**) expression of stemness-related proteins was analyzed via Western blot. * *p* < 0.05; ** *p* < 0.01; *** *p* < 0.001 as compared with control group.

The modulation of autophagy has been a subject of cancer research due to its potential implications in various aspects of cancer progression. A previous study implicated lysosome fusion dysfunction as the main target of the anti-cancer reagent [30]. Here, we employed rapamycin (an autophagy activator) and bafilomycin A1 (a lysosome fusion inhibitor) to investigate their modulatory roles in breast cancer progression. Lysosome fusion dysfunction, induced by bafilomycin A1, enhanced hinokitiol’s effect, leading to the activation of cell death and a reduction in stemness expression. Conversely, the autophagy activator promoted cancer cell progression, minimizing the inhibitory effect of hinokitiol (Figure 7). The results suggest that hinokitiol’s modulation is dependent on the regulation of lysosome fusion.

## 3. Discussion

During breast cancer progression, drug resistance is a major challenge in cancer treatment and is related to the poor survival rates of breast cancer patients [31]. Cancer stem cells possess drug resistance, which is a highly complex process involving poorly characterized pathways. Previous studies have revealed that aggressive and metastatic tumors express stemness-specific markers redundantly [32]. Drawing on various lines of evidence, we formulated a hypothesis that the upregulation of chemoresistance might be facilitated by markers via mechanisms that involve CD44, Nanog, SOX2, and Oct4.

Nanog, a transcription factor that plays a key role in the self-regeneration and maintenance of stemness cells, has also been found in several types of tumor cells [33]. In BCSC, Nanog is activated through a feedback loop mechanism. As a partner of Oct4, Nanog regulates the transcription factor SOX2 to enhance the stemness of breast cancer cells. The transcriptional upregulation of SOX2 and Oct4 by Nanog is, in turn, dependent on an upregulated CD44 [34]. Previous studies demonstrate that hinokitiol could effectively reduce CD133 and Aldehyde dehydrogenase1 (ALDH1) activity and inhibited the cell invasion, migration, and self-renewal progression [35]. CD44 is a cell surface glycoprotein that plays a critical role in cell adhesion, cell migration, and cancer cell invasion. It is also a well-known stem cell marker that has been implicated in stemness regulation and cancer stem cells development [36]. Nanog is involved in cancer cell self-renewal regulation and differentiation, while SOX2 and Oct4 are involved in the maintenance of pluripotency in embryonic stem cells [37]. Previous studies revealed it ability in the modulation of suppressed intracellular aldehyde dehydrogenase (ALDH) activity [38] and vasculogenic mimicry (VM) activity [39] to modulate the stemness progression.

In the present study, we aimed to investigate the effect of hinokitiol on stemness progression in breast cancer cells. To explore how hinokitiol suppress progression, we analyzed the protein expressions of various specific markers in MCF-7, T47D, and MDA-MB-231 cells. Specifically, we determined SOX2, Oct4, Nanog, and CD44 markers. Our results indicated that the expression of SOX2, Oct4, Nanog, and CD44 was highly noticeable in these three cancer cell lines, while treatment with hinokitiol effectively reduced the expression of these stemness-related proteins. This finding suggests that hinokitiol had a potentially promising inhibitory ability against stemness-progression in BC in vitro.

To investigate whether hinokitiol possesses a potent capacity to inhibit the self-renewal abilities of BC cell lines in vitro, we employed a serum-free, ultra-low attachment, non-adherent three-dimensional culture system. This approach allowed for the enrichment of cancer stem-like cells, facilitating our examination of hinokitiol’s impact [40] in the currently used BC cell lines. This attempt provided a conventional three-dimensional cell line model for studying targeted hinokitiol therapy. Experimental confirmation of suspended sphere cells with stem cell-like properties has been demonstrated in various solid tumors including glioma, colon cancer, lung cancer, and ovarian cancer [41,42,43]. In the present study, we cultured three BC cell lines under ultra-low attachment conditions, leading to the formation of an in vitro “three-dimensional microenvironment” composed of BC cell spheres exhibiting “stem cell-like” self-renewal capabilities, which is a key strength of this study. It is noteworthy that these in vitro spheres closely resemble their in vivo counterparts with stem cell-like properties. When treated with hinokitiol for 48 h, there was a significant reduction in both the diameter and number of these spheres (Figure 6). This finding strongly indicates that hinokitiol exerts potent effects in suppressing the self-renewal abilities of breast cancer cells.

The function of autophagy in breast cancer is multifaceted and depends on the type of cancer and the stage of the disease. Autophagy plays a critical role in cancer cell metabolism to support cancer cell survival and proliferation during the TME milieu, therefore, it is essential to focus on clinical trials for cancer targeted-autophagy modulator development [44]. Previous research showed that hinokitiol dramatically increased the protein expression of conversion of LC3B-I to LC3B-II and decreased p62 expression, which indicates autophagic induction in breast cancer cells [26]. Similarly, we observed that hinokitiol also enhance the expression of LC3B II in breast cancer cells, however, there is controversy regarding p62. In our data, the protein expression of p62 was upregulated by hinokitiol, which may be related to the accumulation of p62. This phenomenon was similar to the neferine-activated anti-tumor properties [45] and our previous study of isoliquiritigenin [30] in breast cancer. The disruption of lysosome fusion and autophagolysosome formation, along with inhibition of autophagy influx, leads to p62 accumulation, thereby activating caspase-8 and ultimately resulting in tumor cell death. [30]. Blocking autophagy during hinokitiol treatment enhances apoptosis progression and reduces cancer stem-like cells. Presenting a novel discovery, our study sheds light on the role of autophagy in hinokitiol-modulated stemness progression.

Hinokitiol treatment suppressed the key TFs gene regulator(s) of cancer cell stemness with modulation of the BC stem-like cell lines capacity for self-renewal and cancer cell differentiation during cancer progression (stemness-progression). Therefore, based on our current results, hinokitiol could be used as a potential autophagy-inducer during BC treatment. The induction of apoptosis (cleaved-PARP-1) as well as autophagy (LC3B and p62) via rescuing p-p53 in BC cell lines, inhibiting tumor cell growth, and thence, leading to tumor cell death. p53, identified as a nuclear transcription factor, is a frequently mutated gene in various cancers, and is particularly evident in T47D and MDA-MB-231 triple-negative breast cancer cells [46]. Despite its association with cancer mutations, p53 plays a crucial role in triggering apoptosis. When mutated, p53 can be stabilized through heightened phospholipase D activity [47]. The significance of p53 in MDA-MB-231 cells extends to its involvement in p53-dependent cell cycle arrest and apoptosis, as exemplified by the actions of 6-Gingerol [48]. Hinokitiol led to tumor spheroids shrinkage as inhibited BC stem-like cells self-renewable capacity, suppressed tumor stemness-progression via inhibiting CD44/Nanog/SOX2/Oct4 signaling pathways.

## 4. Materials and Methods

### 4.1. Cell Culture

Breast cancer cell line MCF-7, T47D, and MDA-MB-231 were obtained from the American Type Culture Collection (ATCC; Manassas, VA, USA). MCF-7 cells were cultured in Minimum Essential Medium (MEM, Thermo Fisher Scientific, Waltham, MA, USA) supplemented with 10% fetal bovine serum (FBS, Corning, NY, USA) and 1% penicillin-streptomycin (Corning, NY, USA). T47D cells were maintained in Dulbecco’s modified Eagle’s medium (DMEM) supplemented with 10% FBS and 1% penicillin-streptomycin. MDA-MB-231 cells were cultured in Dulbecco’s Modified Eagle’s Medium (DMEM)/F12 medium (CASSION, Taichung City, Taiwan) supplemented with 10% FBS and 1% penicillin-streptomycin. All cell lines were maintained at 37 °C in a humidified atmosphere with 5% CO_2_. The temperature and CO_2_ concentration were optimized to ensure optimal cell growth and proliferation. The cell lines were routinely monitored for growth and morphology, and the medium was changed every 2–3 days.

To passage the cell lines, they were first washed with phosphate-buffered saline (PBS) to remove any residual medium and debris. Then, trypsin-EDTA (0.25%, Corning) was added to detach the cells from the culture dish. The cell suspension was collected and centrifuged at 1200 rpm for 5 min, and the supernatant was aspirated. The cell pellet was resuspended in fresh growth medium and seeded into new culture dishes.

### 4.2. Reagents

Hinokitiol was purchased from Sigma-Aldrich (St. Louis, MO, USA) and dimethyl sulfoxide (DMSO) was purchased from ECHO Chemical Co. Ltd. (Taipei, Taiwan). Hinokitiol is dissolved in DMSO in 100 μM concentration. The final DMSO concentration was less than 0.1% and the highest amount of DMSO was added to the control group. Rapamycin and Bafilomycin A1 (BafA1) were obtained from Cayman Chemical (Ann Arbor, MI, USA).

### 4.3. MTT Assay

Thiazolyl Blue (MTT) was purchased from MedChemExpress (MCE, Monmouth Junction, NJ, USA). The powder was dissolved in 5 mg/mL concentration and diluted with cultured medium to make a 1 mg/mL working solution. To evaluate the viability assay, cells were seeded in a 96 well plate and treated with hinokitiol for 24, 48, and 72 h. The cells were then cultured with the MTT working solution for 3 h. After aspirating the solution, the remaining crystals were dissolved in dimethyl sulfoxide (DMSO, ECHO Chemical Co., Ltd., Taipei, Taiwan). A VERSA Max microplate reader (Molecular Devices, San Jose, CA, USA) was used to measure the absorbance at 570 and 630 nm wavelengths.

### 4.4. Apoptosis Percentage

An automated fluorescence cell counter (NanoEnTek, Seoul, Republic of Korea) was used to count stained cells. After treating the cells with hinokitiol for 48 h, they were harvested and suspended in 1 mL of phosphate-buffered saline (PBS). Following manufacture instruction, Accustain solution N (NanoEnTek, Republic of Korea) was then used to calculate the number of apoptotic cells, while Accustain solution T was used to calculate the total number of cells. The Accustain solutions are commonly used for the detection of apoptotic cells and can accurately distinguish between apoptotic and necrotic cells.

To perform cell counting, the cell suspension was mixed with the staining solution and loaded into the Accu-Chip 4X (NanoEnTek, Republic of Korea). This chip is a microfluidic device that is designed to count cells using a fluorescence-based detection method. The Accu-Chip 4X can count up to four samples simultaneously, allowing for a high-throughput analysis of multiple samples.

The Accu-Chip 4X was then loaded into the NanoEnTek automated fluorescence cell counter, and the cell counts were obtained. The equipment uses a combination of fluorescence and imaging technologies to count the cells and generate accurate and reliable results.

### 4.5. Sphere Formation

Modified by previous studies [49], after hinokitiol treatment for 48 h, cells were harvest and seeded in the ultra-low attachment plate (CORNING) by using tumorsphere medium (serum free medium suppled with B27, insulin (Life Technologies, Gaithersburg, MD, USA), fibroblast growth factor, epidermal growth factor, Bovine Serum Albumin (Sigma-Aldrich, St. Louis, MO, USA)). The upper and lower edges were sealed with laboratory tape. The plates were incubated for one week without changing or adding medium to allow for tumorsphere formation. After one week, the number of tumorspheres was counted using microscope (Olympus (Tokyo, Japan)). Results were presented as the percentage of tumorspheres formed divided by the initial number of cells seeded.

### 4.6. Western Blot

The radioimmunoprecipitation assay (RIPA) buffer-supplied with protease inhibitor and phosphatase inhibitor (Roche Mannheim, Baden-Württemberg, Germany) were used to homogenize cell pellets. The resulting lysates were loaded onto 10–15% SDS-PAGE gels for protein separation, which was then transferred to PVDF membranes. Antibodies PARP (Cell signaling (Danvers, MA, USA)), p-p53 (Cell signaling), LC3B (Cell signaling), p62 (Cell signaling), CD44 (Proteintech, Rehovot, Israel), Nanog (Proteintech), Oct4 (Proteintech), SOX2 (Proteintech), and glyceraldehyde-3-phosphate dehydrogenase (GAPDH) (Proteintech) were used for blot incubation at a 1:1000 or 1: 10,000 dilutions, followed by incubation with HRP-conjugated anti-rabbit in TBST (1: 10,000, Jackson ImmunoResearch Laboratories (West Grove, PA, USA)). The protein signal was visualized using ECL (T-Pro Biotechnology, New Taipei City, Taiwan) and detected by eBlot Touch Imager (eBlot Photoelectric Technology, Shanghai, China). Analysis was carried out using ImageJ software version 1.54f (NIH, Bethesda, MD, USA).

### 4.7. Statistical Analysis

The data is presented as mean ± standard deviation (SD). All experiments were conducted in independent experiments at least three times in triplicates. Statistical analyses were performed using GraphPad Prism version 8.0 (GraphPad, San Diego, CA, USA). We used Student’s *t*-test, one-way analysis of variance (ANOVA), and Tukey’s post hoc test for analysis. A *p*-value less than 0.05 was considered statistically significant.

## 5. Conclusions

Hinokitiol treatment has demonstrated significant anti-cancer effects against breast cancer (BC) cell lines in vitro. The observed effects include targeting cancer cell proliferation, inducing apoptosis, disrupting autophagy, and inhibiting cancer cell stemness progression, particularly at the dosage of 50 μM. Moreover, hinokitiol exhibited the capacity to reduce BC stem-cell-like self-renewable capabilities.

These findings contribute valuable insights into the potential molecular mechanisms of action underlying hinokitiol’s anti-cancer properties. Its ability to simultaneously induce apoptosis, impair autophagy, and inhibit cancer cell proliferation suggests a multifaceted therapeutic role for hinokitiol in BC treatment. A summarized graphical abstract visually captures these key aspects of hinokitiol’s effects.

The identification of hinokitiol as a potential anti-cancer agent for BC instills hope for the repurposing of safe, effective, sustainable, and natural compounds as therapies for this challenging disease. The emphasis on the green and natural characteristics of hinokitiol further underscores its potential as a promising therapeutic option.

However, it is crucial to acknowledge that these promising in vitro results necessitate further investigation to assess hinokitiol’s efficacy in vivo. Since the dosage did not consistently show effectiveness across different indicators, potential toxic side effects and an overall safety profile need to be carefully evaluated. Comprehensive pre-clinical and clinical studies are imperative to validate the translational potential of hinokitiol as a therapeutic agent for breast cancer.

## Data Availability

Data presented in this study are available upon request from the corresponding author.

## Supplementary Materials

The following supporting information can be downloaded at: https://www.mdpi.com/article/10.3390/ijms25073904/s1.
