# Supplementary material for: Hinokitiol Inhibits Breast Cancer Cells In Vitro Stemness-Progression and Self-Renewal with Apoptosis and Autophagy Modulation via the CD44/Nanog/SOX2/Oct4 Pathway
Source: Int J Mol Sci. 2024 Mar 31;25(7):3904. doi: 10.3390/ijms25073904 (PMC11011552; doi:10.3390/ijms25073904)
Supplement: Supplementary file 1 [file ijms-25-03904-s001.zip › ijms-2882451-supplementary.pdf]

Supplemental Materials and Methods

Flow cytometry measurement for apoptosis

Cell (1×10^5 cells) were seeded in 6 cm dish, treated with hinokitiol for 48 hours. Used Annexin V-FITC apoptosis detection kit (BD Biosciences, Franklin Lakes, NJ, USA) and performed according manufacturer's instructions. Briefly, after harvested the cell, cell was resuspended with binding buffer and stained with 2.5μL of Annexin V-FITC and 2.5μL of PI for 15 min at room temperature. Cell apoptosis distributions was detected by Attune™ NxT Flow Cytometer (Thermo Fisher Scientific, Waltham, MA, USA). The late apoptosis indicated the Annexin V-FITC positive and PI positive percentage, while early apoptosis indicated the Annexin V-FITC positive alone group.

Western blot

The radioimmunoprecipitation assay (RIPA) buffer-supplied with protease inhib-itor and phosphatase inhibitor (Roche Mannheim, Baden-Württemberg, Germany) were used to homogenize cell pellets. The resulting lysates were loaded onto 15% SDS-PAGE gels for protein separation, which was then transferred to PVDF mem-branes. Antibodies Cathepsin B (Cell signaling (Danvers, MA, USA)), and glyceralde-hyde-3-phosphate dehydrogenase (GAPDH) (Proteintech,) against were used for blot incubation at a 1:1000 or 1:10000 dilutions, followed by incubation with HRP-conjugated anti-rabbit in TBST (1:10000, Jackson ImmunoResearch Laboratories (West Grove, PA, USA)). The protein signal was visualized using ECL (T-Pro Biotech-nology, New Taipei City, Taiwan) and detected by eBlot Touch Imager (eBlot Photoe-lectric Technology, Shanghai, China). Analysis was carried out using ImageJ software (NIH, Bethesda, MD, USA).

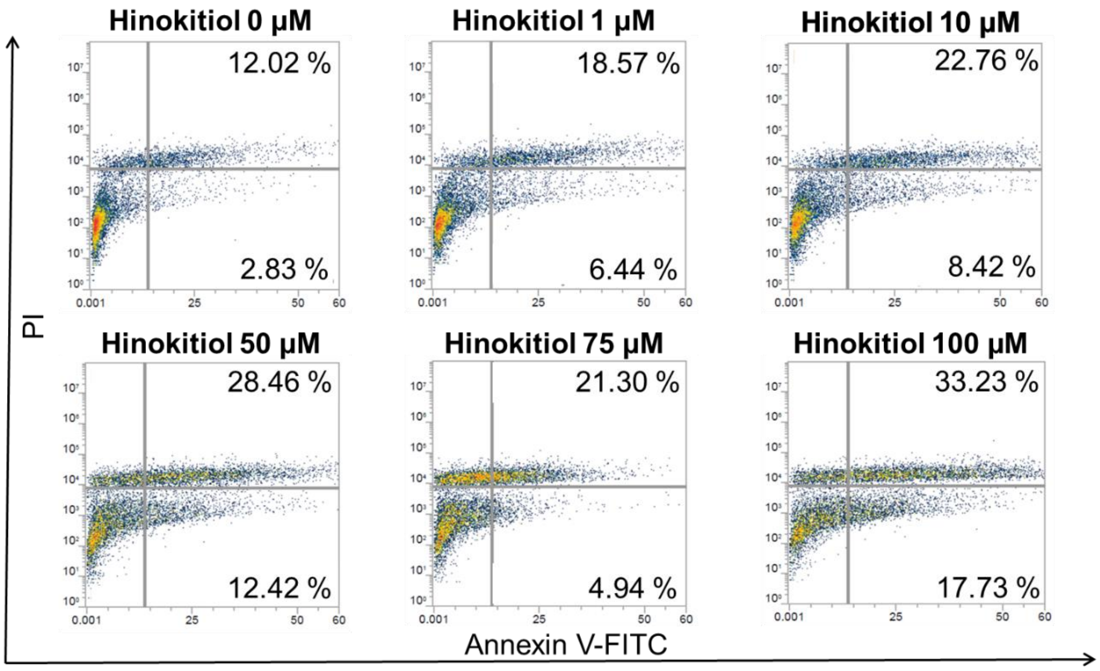

Supplementary Figure S1 Hinokitiol-Induced Apoptosis Activation in MDA-MB-231 Cells

MDA-MB-231 cells were treated with hinokitiol for a duration of 48 hours. After treatment, the cells were stained with Annexin V-FITC and propidium iodide (PI), and flow cytometry was employed to quantify the percentage of cells undergoing apoptosis.

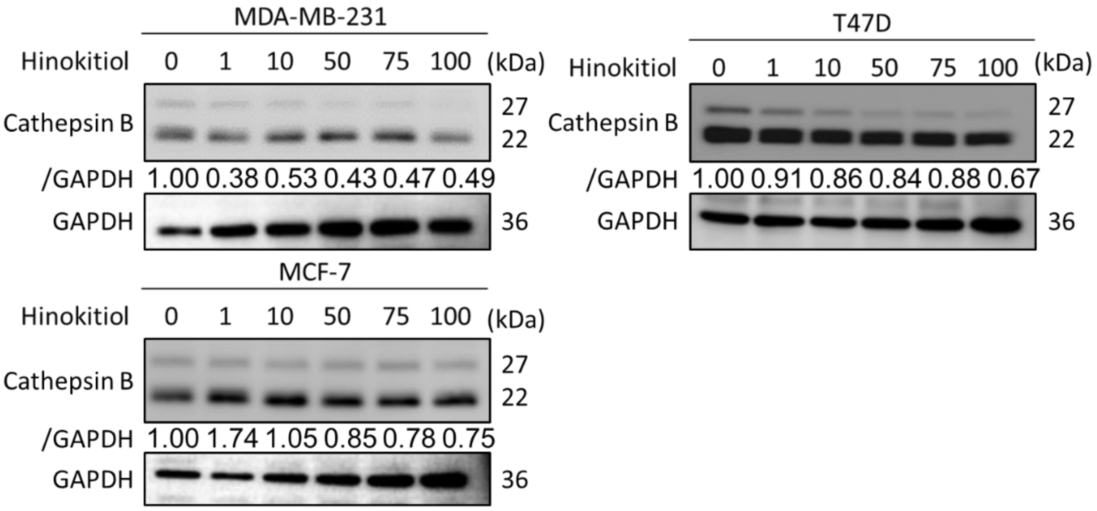

Supplementary Figure S2 Hinokitiol-Induced lysosome reduction in BC cell line.

MDA-MB-231, MCF-7, T47D cells were culture in 6 cm dish and treated with hinokitiol for 48H. Used western blot to evaluate the lysosome marker cathepsin B protein expression.
